# Our Success in the House

**Published:** 1900-12

**Authors:** 


### OUR SUCCESS IN THE HOUSE.

As we go to press with this number the success of Army Legislation seems assured. After a determined battle in the House, the discovery of traitors among those upon whom we depended, by a vote of 80 to 72, the army bill with the veterinary corps as a part of the same was passed. Those who so earnestly supported and carried it through the Senate last year are to-day as zealous in its support as ever, and time has only made them more fully realize its need and importance. While there are new faces in the Senate from several States, they come as new friends of the profession, and the great work of organization of its friends and supporters all over the land will prove the value of this plan of campaign.

---

We are glad to be able to announce that the fire at the New York State Veterinary College was not so serious as at first reported, and will not interfere with the progress of the college course. The losses, while serious, will not be beyond a prompt and complete restoration.

